# Supplementary material for: Causes, characteristics, and patterns of prolonged unplanned school closures prior to the COVID-19 pandemic—United States, 2011–2019
Source: PLoS One. 2022 Jul 29;17(7):e0272088. doi: 10.1371/journal.pone.0272088 (PMC9337642; doi:10.1371/journal.pone.0272088)
Supplement: S1 Table — a PUSC is defined as a school closure lasting ≥5 school days, excluding any scheduled days off. Cumulative incidence of PUSC per 100 schools was computed by dividing the number of schools with PUSCs during the study period (2011–2019) by the total number of all K-12 schools and then multiplying by 100. b Schools were counted each time they experienced a PUSC across the 8-year study period. c Schools with multiple PUSCs were counted only once across the 8-year study period. d The total number of K-12 schools was obtained from the National Center for Education Statistics (NCES) by summing the number of schools reported for each academic year (2011–2012 through 2018–2019) and dividing by eight. e Grade span was not specified for 189 PUSCs, across 186 unique schools. f Urbanicity was not specified for 62 schools. g Regions of the United States Department of Health & Human Services (HHS). https://www.hhs.gov/about/agencies/regional-offices/index.html. (DOCX) [file pone.0272088.s001.docx]

S1 Table. Cumulative incidence of prolonged unplanned school closure (PUSC) per 100 schools, United States, 2011–2019^a^.

|  | **Total Schools Closed,**  **n (cumulative incidence)** | **Unique Schools Closed,**  **n (cumulative incidence)** | **Number of all K-12 schools**^d^ |
| --- | --- | --- | --- |
| **Total** | 22,112 (18.7) | 19,582 (16.6) | 118,153 |
| **Grade level^e^** |  |  |  |
| Elementary school | 9,203 (22.9) | 7,958 (19.8) | 40,174 |
| Elementary to middle school | 4,405 (14.8) | 4,043 (13.5) | 29,853 |
| Elementary to high school | 584 (6.4) | 546 (6.0) | 9,133 |
| Middle school | 3,072 (24.2) | 2,683 (21.1) | 12,703 |
| Middle to high school | 1,021 (14.3) | 946 (13.3) | 7,129 |
| High school | 3,638 (20.5) | 3,220 (18.1) | 17,788 |
| **School Type** |  |  |  |
| Public | 21,784 (22.9) | 19,254 (20.3) | 95,045 |
| Private | 328 (1.4) | 328 (1.4) | 23,109 |
| **Urbanicity^f^** |  |  |  |
| City | 7,682 (22.7) | 7,294 (21.6) | 33,793 |
| Suburban | 7,402 (19.3) | 6,721 (17.5) | 38,354 |
| Town | 2,089 (14.3) | 1,658 (11.3) | 14,649 |
| Rural | 4,877 (15.6) | 3,847 (12.3) | 31,277 |
| **HHS region^g^** |  |  |  |
| HHS 1 | 1,021 (16.9) | 856 (14.2) | 6,029 |
| HHS 2 | 3,150 (32.7) | 3,124 (32.4) | 9,628 |
| HHS 3 | 3,109 (30.1) | 2,352 (22.8) | 10,313 |
| HHS 4 | 6,944 (35.2) | 5,852(29.6) | 19,738 |
| HHS 5 | 1,643 (7.5) | 1,638 (7.5) | 21,825 |
| HHS 6 | 3,163 (20.0) | 2,886 (18.3) | 15,796 |
| HHS 7 | 418 (6.0) | 358 (5.1) | 6,996 |
| HHS 8 | 20 (0.3) | 19 (0.3) | 5,804 |
| HHS 9 | 1,614 (9.9) | 1,561 (9.6) | 16,262 |
| HHS 10 | 1,030 (17.9) | 936 (16.2) | 5,762 |

^a^ PUSC is defined as a school closure lasting ≥5 school days, excluding any scheduled days off. Cumulative incidence of PUSC per 100 schools was computed by dividing the number of schools with PUSCs during the study period (2011-2019) by the total number of all K-12 schools and then multiplying by 100.

^b^ Schools were counted each time they experienced a PUSC across the 8-year study period.

^c^ Schools with multiple PUSCs were counted only once across the 8-year study period.

^d^ The total number of K-12 schools was obtained from the National Center for Education Statistics (NCES) by summing the number of schools reported for each academic year (2011-2012 through 2018-2019) and dividing by eight.

^e^ Grade span was not specified for 189 PUSCs, across 186 unique schools.

^f^ Urbanicity was not specified for 62 schools.

^g^ Regions of the United States Department of Health & Human Services (HHS). <https://www.hhs.gov/about/agencies/regional-offices/index.html>
