# Supplementary material for: Causes, characteristics, and patterns of prolonged unplanned school closures prior to the COVID-19 pandemic—United States, 2011–2019
Source: PLoS One. 2022 Jul 29;17(7):e0272088. doi: 10.1371/journal.pone.0272088 (PMC9337642; doi:10.1371/journal.pone.0272088)
Supplement: S2 Table — a PUSC is defined as a school closure lasting ≥5 school days, excluding any scheduled days off. b Percentages may not add up to 100%, as they are rounded to the nearest tenth of a percent. c Regions of the United States Department of Health & Human Services (HHS). https://www.hhs.gov/about/agencies/regional-offices/index.html. d Urbanicity was not specified for 5 schools with NCES IDs, and unknown for 57 schools without NCES IDs. (DOCX) [file pone.0272088.s002.docx]

S2 Table. Unique schools by the number of prolonged unplanned school closure (PUSC) events experienced, United States, 2011–2019^a, b^

|  | Unique Schools Closed | Number of Unique Schools Closed with: | | | | |
| --- | --- | --- | --- | --- | --- | --- |
|  |  | Single PUSC | Multiple PUSCs | | | |
|  |  |  | 2 PUSCs | 3 PUSCs | 4 PUSCs | Total (2-4 PUSCs) |
| Total, n (row %) | 19,582 | 17,462 (89.2) | 1,770 (9.0) | 290 (1.5) | 60 (0.0) | 2,120 (10.8) |
| HHS region^c^, n (column %) |  |  |  |  |  |  |
| HHS 1 | 856 (4.4) | 695 (4.0) | 157 (8.9) | 4 (1.4) | 0 (0.0) | 161 (7.6) |
| HHS 2 | 3,124 (16.0) | 3,098 (17.7) | 26 (1.5) | 0 (0.0) | 0 (0.0) | 26 (1.2) |
| HHS 3 | 2,352 (12.0) | 1,805 (10.3) | 355 (20.1) | 174 (60.0) | 18 (30.0) | 547 (25.8) |
| HHS 4 | 5,852 (29.9) | 4,951 (28.4) | 752 (42.5) | 107 (36.9) | 42 (70.0) | 901 (42.5) |
| HHS 5 | 1,638 (8.4) | 1,633 (9.4) | 5 (0.3) | 0 (0.0) | 0 (0.0) | 5 (0.2) |
| HHS 6 | 2,886 (14.7) | 2,609 (15.0) | 277 (15.6) | 0 (0.0) | 0 (0.0) | 277 (13.1) |
| HHS 7 | 358 (1.8) | 302 (1.7) | 52 (2.9) | 4 (1.4) | 0 (0.0) | 56 (2.6) |
| HHS 8 | 19 (0.1) | 18 (0.1) | 1 (0.1) | 0 (0.0) | 0 (0.0) | 1 (0.0) |
| HHS 9 | 1,561 (8.0) | 1,509 (8.6) | 51 (2.9) | 1 (0.3) | 0 (0.0) | 52 (2.5) |
| HHS 10 | 936 (4.8) | 842 (4.8) | 94 (5.3) | 0 (0.0) | 0 (0.0) | 94 (4.4) |
| Urbanicity^d^, n (column %) |  |  |  |  |  |  |
| City | 7,294 (37.3) | 6,941 (39.8) | 315 (17.8) | 37 (12.8) | 0 (0.0) | 352 (16.6) |
| Suburban | 6,721 (34.3) | 6,137 (35.1) | 506 (28.6) | 78 (26.9) | 5 (8.3) | 589 (27.8) |
| Town | 1,658 (8.5) | 1,305 (7.5) | 274 (15.5) | 63 (21.7) | 12 (20.0) | 349 (16.5) |
| Rural | 3,847 (19.7) | 3,022 (17.3) | 674 (38.1) | 111 (38.3) | 43 (71.7) | 828 (39.1) |
| Unknown | 62 (0.3) | 57 (0.3) | 1 (0.1) | 1 (0.3) | 0 (0.0) | 2 (0.1) |

^a^ PUSC is defined as a school closure lasting ≥5 school days, excluding any scheduled days off.

^b^ Percentages may not add up to 100%, as they are rounded to the nearest tenth of a percent.

^c^ Regions of the United States Department of Health & Human Services (HHS). https://www.hhs.gov/about/agencies/regional-offices/index.html

^d^ Urbanicity was not specified for 5 schools with NCES IDs, and unknown for 57 schools without NCES IDs.
