# Supplementary material for: Causes, characteristics, and patterns of prolonged unplanned school closures prior to the COVID-19 pandemic—United States, 2011–2019
Source: PLoS One. 2022 Jul 29;17(7):e0272088. doi: 10.1371/journal.pone.0272088 (PMC9337642; doi:10.1371/journal.pone.0272088)
Supplement: S3 Table — a PUSC is defined as a school closure lasting ≥5 school days, excluding any scheduled days off. b Percentages may not add up to 100%, as they are rounded to the nearest tenth of a percent. c Includes building damage from storm, fire in the building, flood from broken pipe, gas leak, unsafe building structure, vandalism/robbery, rat and roach infestation, ventilation issue. d Includes facility issues, no water/unsafe water, no air conditioning, no heat, no electricity. (DOCX) [file pone.0272088.s003.docx]

S3 Table. Cause subcategories of prolonged unplanned school closures (PUSCs) by academic year, United States, 2011–2019^a,b^

|  | PUSC by Academic Year | | | | | | | | |
| --- | --- | --- | --- | --- | --- | --- | --- | --- | --- |
|  | Total | 2011-12 | 2012-13 | 2013-14 | 2014-15 | 2015-16 | 2016-17 | 2017-18 | 2018-19 |
| Total, n (row%*) | 22,112 | 770  (3.5) | 4,513 (20.4) | 996 (4.5) | 1,498 (6.8) | 2,495 (11.3) | 1,382 (6.3) | 7,215 (32.6) | 3,243 (14.7) |
| Cause of PUSC^a^, n (column %*) |  | | | | | | | | |
| Weather |  |  |  |  |  |  |  |  |  |
| Ice/snow/cold | 6,823  (87.8) | 623  (100.0) | 186  (42.9) | 854  (100.0) | 1,418  (100.0) | 1,712  (81.4) | 85  (24.2) | 493  (92.8) | 1,452  (99.0) |
| Rain (heavy/severe/tropical  storms) | 947  (12.2) | 0  (0.0) | 248  (57.1) | 0  (0.0) | 0  (0.0) | 380  (18.2) | 266  (75.8) | 38  (7.2) | 15  (1.0) |
| Natural disaster |  |  |  |  |  |  |  |  |  |
| Hurricane | 9,554  (91.0) | 5  (38.5) | 3,407  (99.9) | 0  (0.0) | 0  (0.0) | 267  (85.9) | 844  (86.0) | 3,936  (91.1) | 1,095  (77.2) |
| Wildfire | 621  (5.9) | 0  (0.0) | 3  (0.1) | 0  (0.0) | 1  (8.3) | 35 (11.3) | 10  (1.0) | 382 (8.8) | 190  (13.4) |
| Flood (river/creek) | 111  (1.1) | 0  (0.0) | 0  (0.0) | 6  (19.4) | 0  (0.0) | 8  (2.6) | 77  (7.9) | 1  (0.0) | 19  (1.3) |
| Tornado | 103  (1.0) | 8  (61.5) | 0  (0.0) | 25  (80.7) | 5  (41.7) | 1  (0.3) | 50  (5.1) | 0  (0.0) | 14  (1.0) |
| Earthquake | 101  (1.0) | 0  (0.0) | 0  (0.0) | 0  (0.0) | 0  (0.0) | 0  (0.0) | 0  (0.0) | 1  (0.0) | 100  (7.1) |
| Volcanic eruption | 6  (0.1) | 0  (0.0) | 0  (0.0) | 0  (0.0) | 6  (50.0) | 0  (0.0) | 0  (0.0) | 0  (0.0) | 0  (0.0) |
| Budget/teacher strike |  |  |  |  |  |  |  |  |  |
| Teacher strike | 3,263  (99.9) | 97  (100.0) | 649  (99.4) | 0  (0.0) | 21  (100.0) | 61  (100.0) | 9  (100.0) | 2,239  (100.0) | 183  (100.0) |
| No state funding | 4  (0.1) | 0  (0.0) | 4  (0.6) | 0  (0.0) | 0  (0.0) | 0  (0.0) | 0  (0.0) | 0  (0.0) | 0  (0.0) |
| Environmental problem |  |  |  |  |  |  |  |  |  |
| Asbestos | 3  (1.5) | 0  (0.0) | 0  (0.0) | 0  (0.0) | 3  (100.0) | 0  (0.0) | 0  (0.0) | 0  (0.0) | 0  (0.0) |
| Lead | 3  (1.5) | 0  (0.0) | 0  (0.0) | 0  (0.0) | 0  (0.0) | 3  (21.4) | 0  (0.0) | 0  (0.0) | 0  (0.0) |
| Mold | 74  (36.5) | 4  (100.0) | 6 (60.0) | 1  (1.1) | 0  (0.0) | 11  (78.6) | 3  (100.0) | 1  (3.5) | 48  (100.0) |
| Water contaminant | 91  (44.8) | 0  (0.0) | 0 (0.0) | 91  (98.9) | 0  (0.0) | 0  (0.0) | 0  (0.0) | 0  (0.0) | 0  (0.0) |
| Poor air quality | 32  (15.8) | 0  (0.0) | 4 (40.0) | 0  (0.0) | 0  (0.0) | 0  (0.0) | 0  (0.0) | 28  (96.6) | 0  (0.0) |
|  |  |  |  |  |  |  |  |  |  |
| Building/utility problem |  |  |  |  |  |  |  |  |  |
| Building issue^c^ | 52  (46.0) | 5  (15.2) | 3  (50.0) | 1  (11.1) | 11  (64.7) | 8  (66.7) | 6  (50.0) | 8  (80.0) | 10  (71.4) |
| Facilities issue^d^ | 61  (54.0) | 28  (84.9) | 3  (50.0) | 8  (88.9) | 6  (35.3) | 4  (33.3) | 6  (50.0) | 2  (20.0) | 4  (28.6) |
| Illness |  |  |  |  |  |  |  |  |  |
| Influenza/influenza-like  illness | 129  (56.3) | 0  (0.0) | 0  (0.0) | 0  (0.0) | 0  (0.0) | 0  (0.0) | 0  (0.0) | 58  (68.2) | 71  (63.4) |
| Other respiratory illness | 26  (11.4) | 0  (0.0) | 0  (0.0) | 0  (0.0) | 0  (0.0) | 0  (0.0) | 26  (100.0) | 0  (0.0) | 0  (0.0) |
| Gastrointestinal illness | 12  (5.2) | 0  (0.0) | 0  (0.0) | 3  (100.0) | 0  (0.0) | 1  (100.0) | 0  (0.0) | 4  (4.7) | 4  (3.6) |
| Meningitis | 3  (1.3) | 0  (0.0) | 0  (0.0) | 0  (0.0) | 0  (0.0) | 0  (0.0) | 0  (0.0) | 0  (0.0) | 3  (2.7) |
| Unknown illness | 59  (25.8) | 0  (0.0) | 0  (0.0) | 0  (0.0) | 2  (100.0) | 0  (0.0) | 0  (0.0) | 23  (27.1) | 34  (30.4) |
| Violence |  |  |  |  |  |  |  |  |  |
| Actualized violence | 9  (23.7) | 0  (0.0) | 0  (0.0) | 7  (100.0) | 1  (4.0) | 0  (0.0) | 0  (0.0) | 1  (100.0) | 0  (0.0) |
| Violence in the community  – safety precaution | 28  (73.7) | 0  (0.0) | 0  (0.0) | 0  (0.0) | 24  (96.0) | 4  (100.0) | 0  (0.0) | 0  (0.0) | 0  (0.0) |
| Threat | 1  (2.6) | 0  (0.0) | 0  (0.0) | 0  (0.0) | 0  (0.0) | 0  (0.0) | 0  (0.0) | 0  (0.0) | 1  (100.0) |

^a^ PUSC is defined as a school closure lasting ≥5 school days, excluding any scheduled days off.

^b^ Percentages may not add up to 100%, as they are rounded to the nearest tenth of a percent.

^c^ Includes building damage from storm, fire in the building, flood from broken pipe, gas leak, unsafe building structure, vandalism/robbery,

rat and roach infestation, ventilation issue.

^d^ Includes facility issues, no water/unsafe water, no air conditioning, no heat, no electricity.
