# Supplementary material for: Causes, characteristics, and patterns of prolonged unplanned school closures prior to the COVID-19 pandemic—United States, 2011–2019
Source: PLoS One. 2022 Jul 29;17(7):e0272088. doi: 10.1371/journal.pone.0272088 (PMC9337642; doi:10.1371/journal.pone.0272088)
Supplement: S4 Table — a PUSC is defined as a school closure lasting ≥5 school days, excluding any scheduled days off. b Regions of the United States Department of Health & Human Services (HHS). https://www.hhs.gov/about/agencies/regional-offices/index.html. c Percentages may not add up to 100%, as they are rounded to the nearest tenth of a percent. d n (column percentages). e n (row percentages). (DOCX) [file pone.0272088.s004.docx]

S4 Table. Causes of prolonged unplanned school closures ^a^ (PUSCs) by academic year and HHS region^b^, United States, 2011–2019^c^

|  | Total^d^  n (column %) | HHS Regions^e^ | | | | | | | | | |
| --- | --- | --- | --- | --- | --- | --- | --- | --- | --- | --- | --- |
|  |  | HHS 1 | HHS 2 | HHS 3 | HHS 4 | HHS 5 | HHS 6 | HHS 7 | HHS 8 | HHS 9 | HHS 10 |
| Total | 22,112 | 1,021 (4.6) | 3,150 (14.3) | 3,109 (14.1) | 6,944 (31.4) | 1,643 (7.4) | 3,143 (14.3) | 418 (1.9) | 20 (0.1) | 1,614 (7.3) | 1,030 (4.7) |
| Cause of PUSC | | | | | | | | | | | |
| Weather | 7,770 | 712 (9.2) | 107 (1.4) | 2,100 (27.0) | 2,028 (26.1) | 916 (11.8) | 982 (12.6) | 326 (4.2) | 0 (0.0) | 10 (0.1) | 589 (7.6) |
| By academic year |  |  |  |  |  |  |  |  |  |  |  |
| 2011-12 | 623 (8.0) | 474 (76.1) | 24 (3.9) | 0 (0.0) | 0 (0.0) | 0 (0.0) | 0 (0.0) | 0 (0.0) | 0 (0.0) | 0 (0.0) | 125 (20.1) |
| 2012-13 | 434 (5.6) | 159 (36.6) | 0 (0.0) | 0 (0.0) | 13 (3.0) | 4 (0.9) | 254 (58.5) | 4 (0.9) | 0 (0.0) | 0 (0.0) | 0 (0.0) |
| 2013-14 | 854 (11.0) | 0 (0.0) | 1 (0.1) | 39 (4.6) | 294 (34.4) | 166 (19.4) | 187 (21.9) | 146 (17.1) | 0 (0.0) | 0 (0.0) | 21 (2.5) |
| 2014-15 | 1,418 (18.3) | 45 (3.2) | 82 (5.8) | 411 (29.0) | 814 (57.4) | 20 (1.4) | 1 (0.1) | 45 (3.2) | 0 (0.0) | 0 (0.0) | 0 (0.0) |
| 2015-16 | 2,092 (26.9) | 0 (0.0) | 0 (0.0) | 1,392 (66.5) | 314 (15.0) | 6 (0.3) | 321 (15.3) | 0 (0.0) | 0 (0.0) | 1 (0.1) | 58 (2.8) |
| 2016-17 | 351 (4.5) | 0 (0.0) | 0 (0.0) | 11 (3.1) | 4 (1.1) | 0 (0.0) | 211 (60.1) | 33 (9.4) | 0 (0.0) | 7 (2.0) | 85 (24.2) |
| 2017-18 | 531 (6.8) | 34 (6.4) | 0 (0.0) | 131 (24.7) | 229 (43.1) | 132 (24.9) | 2 (0.4) | 0 (0.0) | 0 (0.0) | 0 (0.0) | 3 (0.6) |
| 2018-19 | 1,467 (18.9) | 0 (0.0) | 0 (0.0) | 116 (7.9) | 360 (24.5) | 588 (40.1) | 6 (0.4) | 98 (6.7) | 0 (0.0) | 2 (0.1) | 297 (20.3) |
| Natural disaster | 10,496 | 298 (2.8) | 3,018 (28.8) | 115 (1.1) | 4,709 (44.9) | 29 (0.3) | 1,492 (14.2) | 54 (0.5) | 6 (0.1) | 672 (6.4) | 103 (1.0) |
| By academic year |  |  |  |  |  |  |  |  |  |  |  |
| 2011-12 | 13 (0.1) | 1 (7.7) | 3 (23.1) | 1 (7.7) | 6 (46.2) | 2 (15.4) | 0 (0.0) | 0 (0.0) | 0 (0.0) | 0 (0.0) | 0 (0.0) |
| 2012-13 | 3,410 (32.5) | 297 (8.7) | 3,015 (88.4) | 85 (2.5) | 10 (0.3) | 0 (0.0) | 0 (0.0) | 0 (0.0) | 0 (0.0) | 0 (0.0) | 3 (0.1) |
| 2013-14 | 31 (0.3) | 0 (0.0) | 0 (0.0) | 0 (0.0) | 0 (0.0) | 21 (67.7) | 0 (0.0) | 4 (12.9) | 6 (19.4) | 0 (0.0) | 0 (0.0) |
| 2014-15 | 12 (0.1) | 0 (0.0) | 0 (0.0) | 0 (0.0) | 0 (0.0) | 0 (0.0) | 5 (41.7) | 0 (0.0) | 0 (0.0) | 7 (58.3) | 0 (0.0) |
| 2015-16 | 311 (3.0) | 0 (0.0) | 0 (0.0) | 0 (0.0) | 267 (85.9) | 6 (1.9) | 3 (1.0) | 0 (0.0) | 0 (0.0) | 35 (11.3) | 0 (0.0) |
| 2016-17 | 981 (9.4) | 0 (0.0) | 0 (0.0) | 0 (0.0) | 890 (90.7) | 0 (0.0) | 4 (0.4) | 31 (3.2) | 0 (0.0) | 56 (5.7) | 0 (0.0) |
| 2017-18 | 4,320 (41.2) | 0 (0.0) | 0 (0.0) | 0 (0.0) | 2,457 (56.9) | 0 (0.0) | 1,480 (34.3) | 0 (0.0) | 0 (0.0) | 383 (8.9) | 0 (0.0) |
| 2018-19 | 1,418 (13.5) | 0 (0.0) | 0 (0.0) | 29 (2.1) | 1,079 (76.1) | 0 (0.0) | 0 (0.0) | 19 (1.3) | 0 (0.0) | 191 (13.4) | 100 (7.1) |
| Budget/teacher strike | 3,263 | 0 (0.0) | 0 (0.0) | 716 (21.9) | 0 (0.0) | 690 (21.2) | 654 (20.0) | 0 (0.0) | 0 (0.0) | 910 (27.9) | 293 (9.0) |
| By academic year |  |  |  |  |  |  |  |  |  |  |  |
| 2011-12 | 97 (3.0) | 0 (0.0) | 0 (0.0) | 1 (1.0) | 0 (0.0) | 0 (0.0) | 0 (0.0) | 0 (0.0) | 0 (0.0) | 0 (0.0) | 96 (99.0) |
| 2012-13 | 653 (20.0) | 0 (0.0) | 0 (0.0) | 0 (0.0) | 0 (0.0) | 653 (100.0) | 0 (0.0) | 0 (0.0) | 0 (0.0) | 0 (0.0) | 0 (0.0) |
| 2013-14 | 0 (0.0) | 0 (0.0) | 0 (0.0) | 0 (0.0) | 0 (0.0) | 0 (0.0) | 0 (0.0) | 0 (0.0) | 0 (0.0) | 0 (0.0) | 0 (0.0) |
| 2014-15 | 21 (0.6) | 0 (0.0) | 0 (0.0) | 0 (0.0) | 0 (0.0) | 21 (100.0) | 0 (0.0) | 0 (0.0) | 0 (0.0) | 0 (0.0) | 0 (0.0) |
| 2015-16 | 61 (1.9) | 0 (0.0) | 0 (0.0) | 21 (34.4) | 0 (0.0) | 6 (9.8) | 0 (0.0) | 0 (0.0) | 0 (0.0) | 0 (0.0) | 34 (55.7) |
| 2016-17 | 9 (0.3) | 0 (0.0) | 0 (0.0) | 9 (100.0) | 0 (0.0) | 0 (0.0) | 0 (0.0) | 0 (0.0) | 0 (0.0) | 0 (0.0) | 0 (0.0) |
| 2017-18 | 2,239 (68.6) | 0 (0.0) | 0 (0.0) | 675 (30.2) | 0 (0.0) | 0 (0.0) | 654 (29.2) | 0 (0.0) | 0 (0.0) | 910 (40.6) | 0 (0.0) |
| 2018-19 | 183 (5.6) | 0 (0.0) | 0 (0.0) | 10 (5.5) | 0 (0.0) | 10 (5.5) | 0 (0.0) | 0 (0.0) | 0 (0.0) | 0 (0.0) | 163 (89.1) |
| Environmental problem | 203 | 2 (1.0) | 20 (9.9) | 156 (76.9) | 3 (1.5) | 4 (2.0) | 1 (0.5) | 1 (0.5) | 1 (0.5) | 13 (6.4) | 2 (1.0) |
| By academic year |  |  |  |  |  |  |  |  |  |  |  |
| 2011-12 | 4 (2.0) | 0 (0.0) | 3 (75.0) | 0 (0.0) | 0 (0.0) | 0 (0.0) | 0 (0.0) | 1 (25.0) | 0 (0.0) | 0 (0.0) | 0 (0.0) |
| 2012-13 | 10 (4.9) | 0 (0.0) | 7 (70.0) | 0 (0.0) | 1 (10.0) | 0 (0.0) | 1 (10.0) | 0 (0.0) | 0 (0.0) | 0 (0.0) | 1 (10.0) |
| 2013-14 | 92 (45.3) | 0 (0.0) | 0 (0.0) | 91 (98.9) | 0 (0.0) | 1 (1.1) | 0 (0.0) | 0 (0.0) | 0 (0.0) | 0 (0.0) | 0 (0.0) |
| 2014-15 | 3 (1.5) | 0 (0.0) | 0 (0.0) | 0 (0.0) | 0 (0.0) | 0 (0.0) | 0 (0.0) | 0 (0.0) | 0 (0.0) | 3 (100.0) | 0 (0.0) |
| 2015-16 | 14 (6.9) | 0 (0.0) | 0 (0.0) | 1 (7.1) | 1 (7.1) | 3 (21.4) | 0 (0.0) | 0 (0.0) | 0 (0.0) | 9 (64.3) | 0 (0.0) |
| 2016-17 | 3 (1.5) | 0 (0.0) | 0 (0.0) | 0 (0.0) | 0 (0.0) | 0 (0.0) | 0 (0.0) | 0 (0.0) | 1 (33.3) | 1 (33.3) | 1 (33.3) |
| 2017-18 | 29 (14.3) | 0 (0.0) | 0 (0.0) | 28 (96.6) | 1 (3.5) | 0 (0.0) | 0 (0.0) | 0 (0.0) | 0 (0.0) | 0 (0.0) | 0 (0.0) |
| 2018-19 | 48 (23.7) | 2 (4.2) | 10 (20.8) | 36 (75.0) | 0 (0.0) | 0 (0.0) | 0 (0.0) | 0 (0.0) | 0 (0.0) | 0 (0.0) | 0 (0.0) |
| Building/utility problem | 113 | 9 (8.0) | 5 (4.4) | 16 (14.2) | 3 (2.7) | 2 (1.8) | 22 (19.5) | 3 (2.7) | 12 (10.6) | 4 (3.5) | 37 (32.7) |
| By academic year |  |  |  |  |  |  |  |  |  |  |  |
| 2011-12 | 33 (29.2) | 0 (0.0) | 0 (0.0) | 0 (0.0) | 0 (0.0) | 0 (0.0) | 0 (0.0) | 0 (0.0) | 0 (0.0) | 1 (3.0) | 32 (97.0) |
| 2012-13 | 6 (5.3) | 3 (50.0) | 0 (0.0) | 0 (0.0) | 0 (0.0) | 0 (0.0) | 0 (0.0) | 0 (0.0) | 0 (0.0) | 0 (0.0) | 3 (50.0) |
| 2013-14 | 9 (8.0) | 1 (11.1) | 0 (0.0) | 3 (33.3) | 0 (0.0) | 0 (0.0) | 0 (0.0) | 0 (0.0) | 5 (55.6) | 0 (0.0) | 0 (0.0) |
| 2014-15 | 17 (15.0) | 1 (5.9) | 1 (5.9) | 4 (23.5) | 0 (0.0) | 1 (5.9) | 4 (23.5) | 3 (17.7) | 3 (17.7) | 0 (0.0) | 0 (0.0) |
| 2015-16 | 12 (10.6) | 0 (0.0) | 1 (8.3) | 4 (33.3) | 0 (0.0) | 1 (8.3) | 5 (41.7) | 0 (0.0) | 0 (0.0) | 0 (0.0) | 1 (8.3) |
| 2016-17 | 12 (10.6) | 2 (16.7) | 1 (8.3) | 0 (0.0) | 0 (0.0) | 0 (0.0) | 5 (41.7) | 0 (0.0) | 2 (16.7) | 2 (16.7) | 0 (0.0) |
| 2017-18 | 10 (8.9) | 0 (0.0) | 2 (20.0) | 1 (10.0) | 0 (0.0) | 0 (0.0) | 5 (50.0) | 0 (0.0) | 0 (0.0) | 1 (10.0) | 1 (10.0) |
| 2018-19 | 14 (12.4) | 2 (14.3) | 0 (0.0) | 4 (28.6) | 3 (21.4) | 0 (0.0) | 3 (21.4) | 0 (0.0) | 2 (14.3) | 0 (0.0) | 0 (0.0) |
| Illness | 229 | 0 (0.0) | 0 (0.0) | 0 (0.0) | 200 (87.3) | 2 (0.9) | 11 (4.8) | 11 (4.8) | 0 (0.0) | 4 (1.8) | 1 (0.4) |
| By academic year |  |  |  |  |  |  |  |  |  |  |  |
| 2011-12 | 0 (0.0) | 0 (0.0) | 0 (0.0) | 0 (0.0) | 0 (0.0) | 0 (0.0) | 0 (0.0) | 0 (0.0) | 0 (0.0) | 0 (0.0) | 0 (0.0) |
| 2012-13 | 0 (0.0) | 0 (0.0) | 0 (0.0) | 0 (0.0) | 0 (0.0) | 0 (0.0) | 0 (0.0) | 0 (0.0) | 0 (0.0) | 0 (0.0) | 0 (0.0) |
| 2013-14 | 3 (1.3) | 0 (0.0) | 0 (0.0) | 0 (0.0) | 0 (0.0) | 0 (0.0) | 3 (100.0) | 0 (0.0) | 0 (0.0) | 0 (0.0) | 0 (0.0) |
| 2014-15 | 2 (0.9) | 0 (0.0) | 0 (0.0) | 0 (0.0) | 2 (100.0) | 0 (0.0) | 0 (0.0) | 0 (0.0) | 0 (0.0) | 0 (0.0) | 0 (0.0) |
| 2015-16 | 1 (0.4) | 0 (0.0) | 0 (0.0) | 0 (0.0) | 1 (100.0) | 0 (0.0) | 0 (0.0) | 0 (0.0) | 0 (0.0) | 0 (0.0) | 0 (0.0) |
| 2016-17 | 26 (11.4) | 0 (0.0) | 0 (0.0) | 0 (0.0) | 26 (100.0) | 0 (0.0) | 0 (0.0) | 0 (0.0) | 0 (0.0) | 0 (0.0) | 0 (0.0) |
| 2017-18 | 85 (37.1) | 0 (0.0) | 0 (0.0) | 0 (0.0) | 77 (90.6) | 0 (0.0) | 4 (4.7) | 0 (0.0) | 0 (0.0) | 4 (4.7) | 0 (0.0) |
| 2018-19 | 112 (48.9) | 0 (0.0) | 0 (0.0) | 0 (0.0) | 94 (83.9) | 2 (1.8) | 4 (3.6) | 11 (9.8) | 0 (0.0) | 0 (0.0) | 1 (0.9) |
| Violence | 38 | 0 (0.0) | 0 (0.0) | 6 (15.8) | 1 (2.6) | 0 (0.0) | 1 (2.6) | 23 (60.5) | 1 (2.6) | 1 (2.6) | 5 (13.2) |
| By academic year |  |  |  |  |  |  |  |  |  |  |  |
| 2011-12 | 0 (0.0) | 0 (0.0) | 0 (0.0) | 0 (0.0) | 0 (0.0) | 0 (0.0) | 0 (0.0) | 0 (0.0) | 0 (0.0) | 0 (0.0) | 0 (0.0) |
| 2012-13 | 0 (0.0) | 0 (0.0) | 0 (0.0) | 0 (0.0) | 0 (0.0) | 0 (0.0) | 0 (0.0) | 0 (0.0) | 0 (0.0) | 0 (0.0) | 0 (0.0) |
| 2013-14 | 7 (18.4) | 0 (0.0) | 0 (0.0) | 5 (71.4) | 0 (0.0) | 0 (0.0) | 0 (0.0) | 0 (0.0) | 1 (14.3) | 1 (14.3) | 0 (0.0) |
| 2014-15 | 25 (65.8) | 0 (0.0) | 0 (0.0) | 1 (4.0) | 0 (0.0) | 0 (0.0) | 0 (0.0) | 23 (92.0) | 0 (0.0) | 0 (0.0) | 1 (4.0) |
| 2015-16 | 4 (10.5) | 0 (0.0) | 0 (0.0) | 0 (0.0) | 0 (0.0) | 0 (0.0) | 0 (0.0) | 0 (0.0) | 0 (0.0) | 0 (0.0) | 4 (100.0) |
| 2016-17 | 0 (0.0) | 0 (0.0) | 0 (0.0) | 0 (0.0) | 0 (0.0) | 0 (0.0) | 0 (0.0) | 0 (0.0) | 0 (0.0) | 0 (0.0) | 0 (0.0) |
| 2017-18 | 1 (2.6) | 0 (0.0) | 0 (0.0) | 0 (0.0) | 1 (100.0) | 0 (0.0) | 0 (0.0) | 0 (0.0) | 0 (0.0) | 0 (0.0) | 0 (0.0) |
| 2018-19 | 1 (2.6) | 0 (0.0) | 0 (0.0) | 0 (0.0) | 0 (0.0) | 0 (0.0) | 1 (100.0) | 0 (0.0) | 0 (0.0) | 0 (0.0) | 0 (0.0) |

^a^ PUSC is defined as a school closure lasting ≥5 school days, excluding any scheduled days off.

^b^ Regions of the United States Department of Health & Human Services (HHS). https://www.hhs.gov/about/agencies/regional-offices/index.html

^c^ Percentages may not add up to 100%, as they are rounded to the nearest tenth of a percent.

^d^ n (column percentages).

^e^ n (row percentages).
