# Supplementary material for: Causes, characteristics, and patterns of prolonged unplanned school closures prior to the COVID-19 pandemic—United States, 2011–2019
Source: PLoS One. 2022 Jul 29;17(7):e0272088. doi: 10.1371/journal.pone.0272088 (PMC9337642; doi:10.1371/journal.pone.0272088)
Supplement: S5 Table — a PUSC is defined as a school closure lasting ≥5 school days, excluding any scheduled days off. b An additional 17 states experienced a total of 256 (2.4%) natural disaster-related PUSCs and percentages are rounded to the nearest tenth of a percent, therefore percentages may not add up to 100%. (DOCX) [file pone.0272088.s005.docx]

S5 Table. Top ten states with natural disaster-related prolonged unplanned school closures (PUSCs ) by type of natural disaster, United States, 2011–2019^a,b^.

|  | Total | Cause of PUSC (column %) | | | | | |
| --- | --- | --- | --- | --- | --- | --- | --- |
|  |  | Hurricane | Wildfire | Flood | Tornado | Earthquake | Volcanic eruption |
| Total, n (row %) | 10,496 | 9,554 (91.0) | 621 (5.9) | 111 (1.1) | 103 (1.0) | 101 (1.0) | 6 (0.1) |
| Top ten states,  n (column %) |  |  |  |  |  |  |  |
| Florida | 2,409 (23.0) | 2,409 (25.2) | 0 (0.0) | 0 (0.0) | 0 (0.0) | 0 (0.0) | 0 (0.0) |
| New York | 1.997 (19.0) | 1,997 (21.0) | 0 (0.0) | 0 (0.0) | 0 (0.0) | 0 (0.0) | 0 (0.0) |
| Texas | 1,491 (14.2) | 1,480 (15.5) | 0 (0.0) | 2 (1.8) | 9 (8.7) | 0 (0.0) | 0 (0.0) |
| North Carolina | 1,106 (10.5) | 1,106 (11.6) | 0 (0.0) | 0 (0.0) | 0 (0.0) | 0 (0.0) | 0 (0.0) |
| New Jersey | 1,021 (9.7) | 1,021 (10.7) | 0 (0.0) | 0 (0.0) | 0 (0.0) | 0 (0.0) | 0 (0.0) |
| South Carolina | 776 (7.4) | 776 (8.1) | 0 (0.0) | 0 (0.0) | 0 (0.0) | 0 (0.0) | 0 (0.0) |
| California | 663 (6.3) | 0 (0.0) | 618 (99.5) | 45 (40.5) | 0 (0.0) | 0 (0.0) | 0 (0.0) |
| Georgia | 380 (3.6) | 334 (3.5) | 0 (0.0) | 0 (0.0) | 46 (44.7) | 0 (0.0) | 0 (0.0) |
| Connecticut | 297 (2.8) | 297 (3.1) | 0 (0.0) | 0 (0.0) | 0 (0.0) | 0 (0.0) | 0 (0.0) |
| Alaska | 100 (1.0) | 0 (0.0) | 0 (0.0) | 0 (0.0) | 0 (0.0) | 100 (99.0) | 0 (0.0) |

^a^ PUSC is defined as a school closure lasting ≥5 school days, excluding any scheduled days off.

^b^ An additional 17 states experienced a total of 256 (2.4%) natural disaster-related PUSCs and percentages are rounded to the nearest tenth of a percent, therefore percentages may not add up to 100%.
