# Supplementary material for: Causes, characteristics, and patterns of prolonged unplanned school closures prior to the COVID-19 pandemic—United States, 2011–2019
Source: PLoS One. 2022 Jul 29;17(7):e0272088. doi: 10.1371/journal.pone.0272088 (PMC9337642; doi:10.1371/journal.pone.0272088)
Supplement: S6 Table — a PUSC is defined as a school closure lasting ≥5 school days, excluding any scheduled days off. b Regions of the United States Department of Health & Human Services (HHS). https://www.hhs.gov/about/agencies/regional-offices/index.html. C Percentages may not add up to 100%, as they are rounded to the nearest tenth of a percent. (DOCX) [file pone.0272088.s006.docx]

S6 Table. Seasonality of weather-related, prolonged unplanned school closures ^a^ (PUSCs) by HHS Region^b^, United States, 2011–2019^c^

|  | Total | HHS Regions | | | | | | | | | |
| --- | --- | --- | --- | --- | --- | --- | --- | --- | --- | --- | --- |
|  |  | HHS 1 | HHS 2 | HHS 3 | HHS 4 | HHS 5 | HHS 6 | HHS 7 | HHS 8 | HHS 9 | HHS 10 |
| Total weather-related PUSCs, n (row %) | 7,770 | 712 (9.2) | 107 (1.4) | 2,100 (27.0) | 2,028 (26.1) | 916 (11.8) | 982 (12.6) | 326 (4.2) | 0  (0.0) | 10 (0.1) | 589 (7.6) |
| Season of weather-related PUSCs, n (column %) |  |  |  |  |  |  |  |  |  |  |  |
| Fall | 711 (9.2) | 496 (69.7) | 106 (99.1) | 1  (0.1) | 0  (0.0) | 4  (0.4) | 8  (0.8) | 31  (9.5) | 0  (0.0) | 7  (70.0) | 58  (9.9) |
| Winter | 6,060  (78.0) | 204  (28.7) | 1  (0.9) | 2,088  (99.4) | 1,967  (97.0) | 908  (99.1) | 199  (20.3) | 284  (87.1) | 0  (0.0) | 2  (20.0) | 407  (69.1) |
| Spring | 523  (6.7) | 12  (1.7) | 0  (0.0) | 0  (0.0) | 60  (3.0) | 4  (0.4) | 321  (32.7) | 9  (2.8) | 0  (0.0) | 1  (10.0) | 116  (19.7) |
| Summer | 476  (6.1) | 0  (0.0) | 0  (0.0) | 11  (0.5) | 1  (0.1) | 0  (0.0) | 454  (46.2) | 2  (0.6) | 0  (0.0) | 0  (0.0) | 8  (1.4) |

^a^ PUSC is defined as a school closure lasting ≥5 school days, excluding any scheduled days off.

^b^ Regions of the United States Department of Health & Human Services (HHS). https://www.hhs.gov/about/agencies/regional-offices/index.html

^C^ Percentages may not add up to 100%, as they are rounded to the nearest tenth of a percent.
