# Supplementary material for: Causes, characteristics, and patterns of prolonged unplanned school closures prior to the COVID-19 pandemic—United States, 2011–2019
Source: PLoS One. 2022 Jul 29;17(7):e0272088. doi: 10.1371/journal.pone.0272088 (PMC9337642; doi:10.1371/journal.pone.0272088)
Supplement: S7 Table — a PUSC is defined as a school closure lasting ≥5 school days, excluding any scheduled days off. b Percentages may not add up to 100%, as they are rounded to the nearest tenth of a percent. c The closure announcement did not specify the type(s) of illness. (DOCX) [file pone.0272088.s007.docx]

S7 Table. States with illness-related prolonged unplanned school closures (PUSCs ) by illness category, United States, 2011–2019^a,b^.

|  | Total | Cause of PUSC | | | |
| --- | --- | --- | --- | --- | --- |
|  |  | Influenza/influenza- like illness | Gastrointestinal illness | Meningitis | Unknown illness^c^ |
| Total, n (row %) | 229 | 155 (67.7) | 12 (5.2) | 3 (1.3) | 59 (25.8) |
| State, n (column %) |  |  |  |  |  |
| Kentucky | 120 (52.4) | 95 (61.3) | 0 (0.0) | 0 (0.0) | 25 (42.4) |
| Tennessee | 78 (34.1) | 44 (28.4) | 0 (0.0) | 0 (0.0) | 34 (57.6) |
| Texas | 8 (3.5) | 5 (3.2) | 0 (0.0) | 3 (100.0) | 0 (0.0) |
| Missouri | 7 (3.1) | 7 (4.5) | 0 (0.0) | 0 (0.0) | 0 (0.0) |
| California | 4 (1.8) | 0 (0.0) | 4 (33.3) | 0 (0.0) | 0 (0.0) |
| Iowa | 4 (1.8) | 0 (0.0) | 4 (33.3) | 0 (0.0) | 0 (0.0) |
| Oklahoma | 3 (1.3) | 0 (0.0) | 3 (25.0) | 0 (0.0) | 0 (0.0) |
| Alabama | 2 (0.9) | 1 (0.7) | 1 (8.3) | 0 (0.0) | 0 (0.0) |
| Minnesota | 2 (0.9) | 2 (1.3) | 0 (0.0) | 0 (0.0) | 0 (0.0) |
| Idaho | 1 (0.4) | 1 (0.7) | 0 (0.0) | 0 (0.0) | 0 (0.0) |

^a^ PUSC is defined as a school closure lasting ≥5 school days, excluding any scheduled days off.

^b^ Percentages may not add up to 100%, as they are rounded to the nearest tenth of a percent.

^c^ The closure announcement did not specify the type(s) of illness.
