# Supplementary material for: Causes, characteristics, and patterns of prolonged unplanned school closures prior to the COVID-19 pandemic—United States, 2011–2019
Source: PLoS One. 2022 Jul 29;17(7):e0272088. doi: 10.1371/journal.pone.0272088 (PMC9337642; doi:10.1371/journal.pone.0272088)
Supplement: Attachment — Submitted filename: Response to Reviewers_PONE_04.15.2022.docx [file pone.0272088.s008.docx]

Response to Reviewers

Thank you for the opportunity to revise and strengthen this manuscript. Please find below a point-by-point response to both the academic editor, Dr. Chowell, and the reviewer, Dr. Fung.

Sincerely,

Nicole Zviedrite

**ACADEMIC EDITOR:**

| **Journal requirements** | **Response** |
| --- | --- |
| 1. Please ensure that your manuscript meets PLOS ONE's style requirements, including those for file naming. The PLOS ONE style templates can be found at  <https://journals.plos.org/plosone/s/file?id=wjVg/PLOSOne_formatting_sample_main_body.pdf> and  <https://journals.plos.org/plosone/s/file?id=ba62/PLOSOne_formatting_sample_title_authors_affiliations.pdf> | We have reviewed and edited to ensure the manuscript meets the style requirements and file naming protocols. |
| 2. We note that the grant information you provided in the ‘Funding Information’ and ‘Financial Disclosure’ sections do not match.   When you resubmit, please ensure that you provide the correct grant numbers for the awards you received for your study in the ‘Funding Information’ section. | This study was not funded by any grants.  Thank you for bringing this to our attention. Please see amendments to the ‘Funding Information’ and ‘Financial Disclosure’ sections below.  Please find below the amended funding statement:  This study was supported by the United States Centers for Disease Control and Prevention (http://www.cdc.gov/). The co-authors are or were employees (NZ HG FA AU) or contractors (FJ) of the US CDC at the time of the study. F**erdous Jahan (FJ)** is employed by Cherokee Nation Operational Solutions, LLC. The funder (Cherokee Nation Operational Solutions) provides support in the form of salary for the author (FJ), but did not have any additional role in the study design, data collection and analysis, decision to publish, or preparation of the manuscript. The specific roles of all authors are articulated in the ‘Author Contributions’ section.  Please find below the updated Competing Interests Statement:  Ferdous Jahan is employed by Cherokee Nation Operational Solutions, LLC. This does not alter our adherence to PLOS ONE policies on sharing data and materials. |
| 3. We note that you have stated that you will provide repository information for your data at acceptance. Should your manuscript be accepted for publication, we will hold it until you provide the relevant accession numbers or DOIs necessary to access your data. If you wish to make changes to your Data Availability statement, please describe these changes in your cover letter and we will update your Data Availability statement to reflect the information you provide. | Understood, thank you. We have cleared our data internally and will be posting it if accepted at the time of acceptance. At that time, we will provide the relevant access information for these data. |
| 4. We note that you have included the phrase “data not shown” in your manuscript. Unfortunately, this does not meet our data sharing requirements. PLOS does not permit references to inaccessible data. We require that authors provide all relevant data within the paper, Supporting Information files, or in an acceptable, public repository. Please add a citation to support this phrase or upload the data that corresponds with these findings to a stable repository (such as Figshare or Dryad) and provide and URLs, DOIs, or accession numbers that may be used to access these data. Or, if the data are not a core part of the research being presented in your study, we ask that you remove the phrase that refers to these data. | We have added supplementary tables (Tables S2-S7) to address these gaps and have replaced the phrase “data not shown” with the reference to the relevant table(s). |
| 5. Please include your full ethics statement in the ‘Methods’ section of your manuscript file. In your statement, please include the full name of the IRB or ethics committee who approved or waived your study, as well as whether or not you obtained informed written or verbal consent. If consent was waived for your study, please include this information in your statement as well. | We have added the name of the committee who reviewed the study for human subjects research, and, per your directions, this has been added to the methods section.  As stated in the submission, “The project underwent ethical review at the Centers for Disease Control and Prevention and was determined not to involve human subjects; it was therefore not subject to institutional review board review requirements.” |
| 6. Please review your reference list to ensure that it is complete and correct. If you have cited papers that have been retracted, please include the rationale for doing so in the manuscript text, or remove these references and replace them with relevant current references. Any changes to the reference list should be mentioned in the rebuttal letter that accompanies your revised manuscript. If you need to cite a retracted article, indicate the article’s retracted status in the References list and also include a citation and full reference for the retraction notice. | The reference list has been fully reviewed and updated.   - No papers have been retracted. - One (1) reference was removed, and replaced with the correct reference.   - Removed: Zheteyeva YA, Moro PL, Tepper NK, Rasmussen SA, Barash FE, Revzina NV, et al. Adverse event reports after tetanus toxoid, reduced diphtheria toxoid, and acellular pertussis vaccines in pregnant women. Am J Obstet Gynecol. 2012;207(1):59 e1-7.   - Replacement: Zheteyeva Y, Rainey JJ, Gao H, Jacobson EU, Adhikari BB, Shi J, et al. Unintended costs and consequences of school closures implemented in preparation for Hurricane Isaac in Harrison County School District, Mississippi, August-September 2012. PLoS One. 2017;12(11):e0184326. - Two (2) additional references have been added, addressing reviewer comments to the discussion section on limitations:  1. Jackson AM, Mullican LA, Tse ZTH, Yin J, Zhou X, Kumar D, et al. Unplanned Closure of Public Schools in Michigan, 2015-2016: Cross-Sectional Study on Rurality and Digital Data Harvesting. J Sch Health. 2020;90(7):511-9. 2. Ahweyevu JO, Chukwudebe NP, Buchanan BM, Yin J, Adhikari BB, Zhou X, et al. Using Twitter to Track Unplanned School Closures: Georgia Public Schools, 2015-17. Disaster Med Public Health Prep. 2021;15(5):568-72. |

**REVIEWER 1:**

| **Review 1 comments** | **Response** |
| --- | --- |
| This is an important piece of research. The authors analyzed the "prolonged unplanned school closures" data collected by the CDC’s Community Interventions for Infection Control Unit (CI-ICU) and their contractors via online systematic search. The estimated number of student-days lost (as shown in Table 2: "Top twenty reasons for prolonged unplanned school closures (PUSC) according to the number of student-days lost, United States, 2011-2019") are important information both for education professionals and for disaster preparedness and response professionals. | Thank you for your comment and your thoughtful review. |
| 1) Please clarify the following inconsistency of definition: is it 5d+ or 6d+? I guess – but this is not clear in the Methods section – is that the authors analyzed a subset of the data that contains unplanned school closures of 5d+ long. Among these 5d+ long unplanned school closures, the authors then analyze the association between selected characteristics and school closures of 6d+ using school closures of 5 days as the reference category (as explained in Line 105-108 and in the footnote to Table 3 in Line 217-221). This is interesting. But perhaps you need to better justify why you don’t analyze the whole dataset, or use schools with 0 d (or 0 to 5 d) of unplanned school closures as your reference category (for example). In other words, is it more important to know the adjusted odds ratio between those with unplanned school closures of 6+ d versus 5 d, than to know the adjusted odds ratio between 6+ d and 0 d, or that between 6+ d and 0-5 d? It is better to have a scientific reason to justify your choice of analysis.   • Line 70-71: “A closure duration of ≥5 days was categorized as PUSC.” • Line 98-99: “The number of students enrolled pertains to all schools regardless of whether a school closed or not (i.e., schools with closes of 0 days, 1 – 4 days, or ≥5 days). • Line 105-108: “The association between selected characteristics and school closures of ≥6 days among schools with PUSCs was assessed using schools as the unit of analysis…. The dependent variable in the regression models was coded as 0 (closure of 5 days) and 1 (closure of ≥6 days).” | Thank you for this comment. In the analysis we are showing the AOR between 6+ days versus 5 days, as 6 day is the median closure length for prolonged unplanned school closures. We have added text to justify this decision, and resolve confusion regarding the definition of “prolonged”.  We have chosen not to compare to schools that did not close (0 days of closure) because we would be unable to look at reasons for closure, which shows to be an important factor related to closure duration. |
| 2) In your limitation paragraph, please include an additional limitation: Given the source of data for this dataset (online systematic searches), some unplanned school closures that could have been identified using social media data might have been missed by analyzing online systematic searches alone. As shown in two recent studies that analyzed social media data in addition to the OSS data provided by the CDC’s CI-ICU (i.e., the authors of this manuscript), additional unplanned school closures that were not identified using OSS alone, were then identified on social media posts posted by the schools or school districts. This reviewer is confident that the authors are aware of these papers as they have cross-cleared those papers in the CDC. Perhaps the authors should cite them as well: • Jackson AM, Mullican LA, Tse ZTH, Yin J, Zhou X, Kumar D, Fung ICH (2020). Unplanned closure of public schools in Michigan, 2015-2016: Cross-sectional study on rurality and digital data harvesting. Journal of School Health. 90(7):511-519. DOI: <https://doi.org/10.1111/josh.12901> • Ahweyevu JO, Chukwudebe NP, Buchanan BM, Yin J, Adhikari BB, Zhou X, Tse ZTH, Chowell G, Meltzer MI, Fung ICH (2020). Using Twitter to Track Unplanned School Closures: Georgia Public Schools, 2015-17. Disaster Medicine and Public Health Preparedness. DOI: <https://doi.org/10.1017/dmp.2020.65> | Thank you for this comment. We have added text to further explain where we may be missing closures (those announced via social media or via non-public methods), and included the two citations provided.  With your permission, we would also like to acknowledge your contribution in strengthening our manuscript with your review. |
| Line 60: Please write the full name of the virus when you introduce the acronym. Suggestion: please write: “severe acute respiratory syndrome coronavirus 2 (SARS-CoV-2)” | Thank you for noticing our omission. This has been added. |
| Liner 168: “(73,072,759or 81%)”: There should be space between the number and the word “or”. | Space has been added. |
| Line 326: “categorized as prolonged, (lasting ≥5 days),”: You may delete the comma before “(lasting ≥5 days)”. | Comma has been deleted. |
